# Measurement fidelity, not model complexity, governs individual cognitive trajectory prediction in Parkinson's disease

**DOI:** 10.1038/s41746-026-02681-8

**Published:** 2026-06-06

**Authors:** Wei Lin, Sanjeet S. Grewal

**Affiliations:** Department of Neurologic Surgery, Mayo Clinic, Jacksonville, FL USA

**Keywords:** Biomarkers, Computational biology and bioinformatics, Neurology, Neuroscience

## Abstract

Whether individual-level cognitive trajectories in Parkinson's disease are predictable remains unresolved. Here, we provide convergent evidence that measurement fidelity, rather than model complexity, governs the prediction ceiling. We evaluated ten machine learning paradigm families across 26 configurations in two independent cohorts—the Parkinson’s Progression Markers Initiative (PPMI, *N* = 1018) and the National Alzheimer’s Coordinating Center (NACC, *N* = 523). Motor subtype classification succeeded as a positive control (AUROC = 0.869), while cognitive trajectory prediction from the Montreal Cognitive Assessment remained uninformative (AUROC = 0.581) across all methods, including 42 genetic features. Synthetic experiments confirmed that models recover trajectories at high signal-to-noise ratios but collapse uniformly at levels present in clinical screening data. Replacing coarse screening with detailed neuropsychological tests improved prediction in both cohorts (PPMI: Symbol Digit Modalities Test AUROC = 0.725 vs. MoCA 0.596; NACC: Logical Memory AUROC = 0.684 vs. MMSE 0.648). Within-patient trajectory *R*² = 0.20 for MoCA domains confirmed that approximately 80% of score variance represents non-signal variance. These findings redirect the clinical AI agenda from algorithm development toward measurement innovation: higher-frequency, higher-fidelity cognitive instruments are necessary before individual-level prediction becomes feasible.

## Introduction

Cognitive impairment represents one of the most disabling non-motor features of Parkinson's disease (PD), affecting up to 80% of individuals over the disease course and progressing to dementia in approximately 40% within 10 years of diagnosis^1^. The heterogeneity of cognitive decline trajectories—ranging from decades of stability to rapid deterioration within years—constitutes a major challenge for clinical management^2^. Accurate individual-level prediction of cognitive trajectories would enable personalized monitoring protocols and earlier therapeutic intervention.

The application of advanced machine learning methods to PD progression modeling has accelerated considerably. Tabular foundation models^3^, multi-task architectures including progressive layered extraction^4^ and multi-gate mixture-of-experts^5^, neural ordinary differential equations for irregular time series^6^, graph neural networks for causal discovery^7,8^, and variational autoencoders for representation learning have each been proposed for disease trajectory modeling. Several studies have reported encouraging metrics for deep learning approaches to PD cognitive decline prediction. Recent PPMI-based studies include genetic predictor identification^9^, subtype-based progression modeling^10^, and trajectory prediction from clinical and neuroimaging features^11^.

These studies share an implicit assumption: that cognitive trajectory prediction is fundamentally a “method” problem—that algorithmic sophistication, given sufficient data, will enable individual-level prediction. This assumption has not been systematically tested. An alternative hypothesis is that prediction failure reflects “measurement” limitations—that currently available clinical cognitive instruments lack sufficient fidelity to support individual-level trajectory prediction, regardless of the analytical method applied.

We designed a multi-cohort eliminative study to adjudicate between these hypotheses. We evaluated ten machine learning paradigm families spanning 26 model configurations across four feature modalities in the PPMI cohort (*N* = 1018), with external validation in the NACC PD cohort (*N* = 523). Motor subtype classification served as a positive control. We then provided three layers of convergent evidence for measurement limitation: synthetic signal recovery experiments demonstrating that models succeed at high signal-to-noise ratios, neuropsychological outcome substitution showing that higher-fidelity instruments improve prediction, and cross-cohort replication confirming generalizability.

## Results

### Positive control: motor subtype classification succeeds

**Fig. 1:**
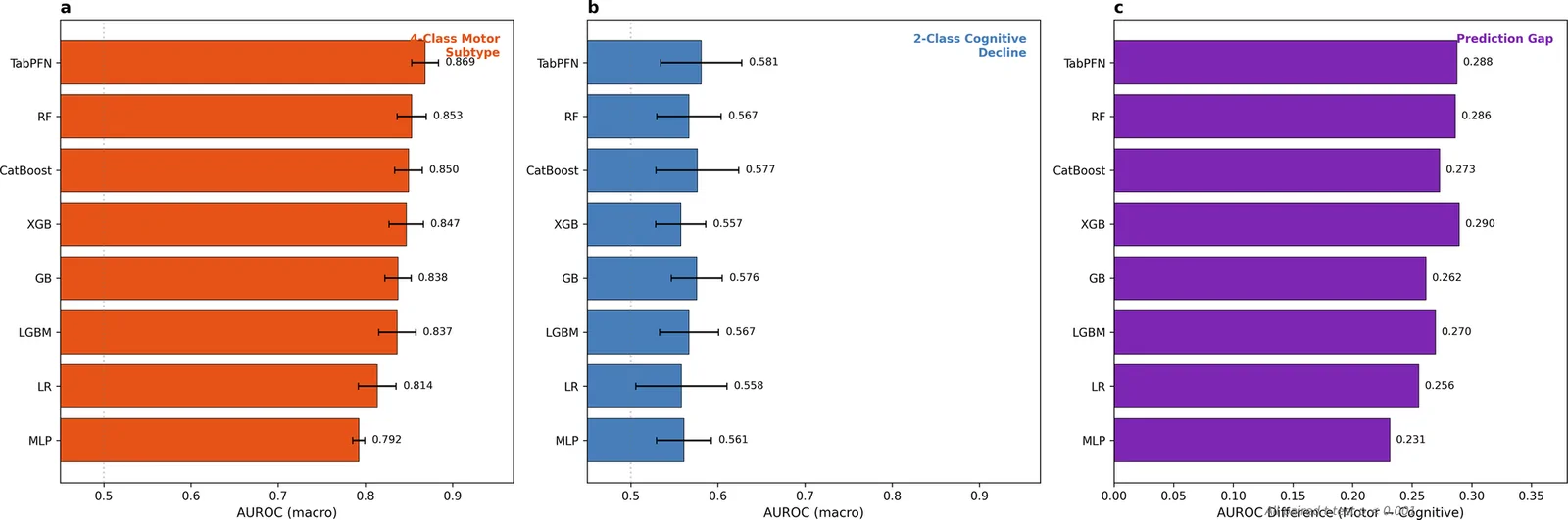
Prediction asymmetry between motor subtype and cognitive trajectory (PPMI, *N* = 1018). **a** AUROC for four-class motor subtype classification across eight models. All models achieve AUROC exceeding 0.79, with TabPFN v2 reaching 0.869. **b** AUROC for two-class cognitive trajectory prediction using the same features and models. All models show clinically uninformative discrimination (AUROC 0.527–0.581). **c** Motor-cognitive AUROC difference for each model. The consistent gap exceeding 0.23 AUROC units across all models (all *p* < 0.001, paired t-test) localizes the prediction barrier to the cognitive target rather than to the analytical approach.

All eight cross-sectional models achieved strong discrimination for the four-class motor subtype target (Table 1 and Fig. 1a). TabPFN v2 achieved the highest AUROC (0.869, SD 0.015), followed by RF (0.853, SD 0.013) and CatBoost (0.850, SD 0.009). All models exceeded AUROC 0.79. This result establishes that the feature matrix contains sufficient information for a meaningful classification task and that the analytical pipeline produces valid discrimination.

**Table 1:** Prediction results: motor subtype vs. cognitive trajectory (PPMI)

Model	Motor subtype AUROC (SD)	Cognitive 2-class AUROC (SD)	Difference
TabPFN v2	0.869 (0.015)	0.581 (0.047)	−0.288
RF	0.853 (0.013)	0.558 (0.032)	−0.295
CatBoost	0.850 (0.009)	0.552 (0.039)	−0.298
XGBoost	0.846 (0.011)	0.552 (0.039)	−0.294
LightGBM	0.845 (0.013)	0.537 (0.030)	−0.308
LR	0.840 (0.015)	0.547 (0.048)	−0.293
GBT	0.828 (0.015)	0.548 (0.028)	−0.280
MLP	0.793 (0.016)	0.527 (0.035)	−0.266
Genetics uplift (GFS)			
GFS1 (clinical, RF)	0.842	0.558	−0.284
GFS4 (all genetics, RF)	0.853	0.559	−0.294
Delta RF (GFS4-GFS1)	+0.011	+0.001	
p-value (RF)	0.108	0.937	
GFS1 (clinical, LightGBM)	0.828	0.548	−0.280
GFS4 (all genetics, LightGBM)	0.851	0.567	−0.284
Delta LightGBM (GFS4-GFS1)	+0.023	+0.019	
p-value (LightGBM)	0.004	0.235	
Neuropsych outcome (PPMI, RF)			
SDMT (processing speed)	--	0.725 (0.017)	--
HVLT delayed recall (episodic memory)	--	0.651 (0.025)	--
LNS (working memory)	--	0.638 (0.022)	--
MoCA PC1 (composite)	--	0.596 (0.032)	--
NACC outcome (RF)			
Logical Memory Immediate	--	0.684 (0.070)	--
MMSE	--	0.648 (0.049)	--
MoCA	--	0.513 (0.119)	--

*AUROC* area under the receiver operating characteristic curv, *SD* standard deviation, *GFS* genetics feature set, *SDMT* symbol digit modalities test, *HVLT* Hopkins verbal learning test, *LNS* letter-number sequencing, *MoCA* Montreal Cognitive Assessment, *MMSE* mini-mental state examination.

Because motor subtype labels were derived from longitudinal UPDRS-III trajectories and baseline UPDRS-III total score is included among the input features, a potential circularity exists. To address this, we performed an ablation analysis under two conditions: Ablated (excluding baseline UPDRS-III total and all subscores) and Strict (excluding all motor assessment variables, including Hoehn and Yahr and Schwab and England ADL). Under the Ablated condition, RF achieved AUROC = 0.8217 (SD 0.0146), a decrease of 0.0303 from the full-feature result (0.8520; paired t-test *p* = 0.0021). Under the Strict condition, RF achieved AUROC = 0.7937 (SD 0.0159), a decrease of 0.0583 (*p* = 0.00033). These drops were consistent across all models (e.g., XGBoost: 0.8451 → 0.8181 → 0.7802; Gradient Boosting: 0.8426 → 0.8100 → 0.7763). Critically, the motor–cognitive AUROC gap remained large under both ablation conditions: 0.2407 (Ablated) and 0.2127 (Strict), both exceeding 0.20, preserving the core argument that cognitive prediction failure is target-specific rather than pipeline-related.

### Systematic method elimination shows cognitive prediction failure is universal

For the two-class cognitive trajectory target, all cross-sectional models yielded AUROC values between 0.527 and 0.581 (Table 1 and Fig. 1b). TabPFN v2 achieved 0.581 (SD 0.047), RF 0.558 (SD 0.032), and LR 0.547 (SD 0.048). No model exceeded AUROC 0.60. A paired t-test confirmed that the motor-cognitive AUROC difference was statistically significant for all eight models (all *p* < 0.001; Fig. 1c). This finding is consistent with previous observations that tree-based methods remain competitive with deep learning on tabular data^12^.

Advanced paradigms fared no better. Progressive layered extraction underperformed independent random forests on all seven MoCA domains (MAE increase 5.7–13.6%). Linear extrapolation achieved Spearman *ρ* = 0.771 across domains, while latent ODE achieved rho near 0 and GRU rho between −0.06 and 0.07. The coupled graph ODE showed identical performance to independent baselines, with all learnable coupling parameters remaining at initialization values. MC²VAE achieved no disentanglement beyond random channel separation (*p* = 0.85 vs. permutation null). These systematic failures across ten paradigm families indicate that no existing method overcomes the cognitive prediction barrier.

### Simulation recovery demonstrates signal limitation

**Fig. 2:**
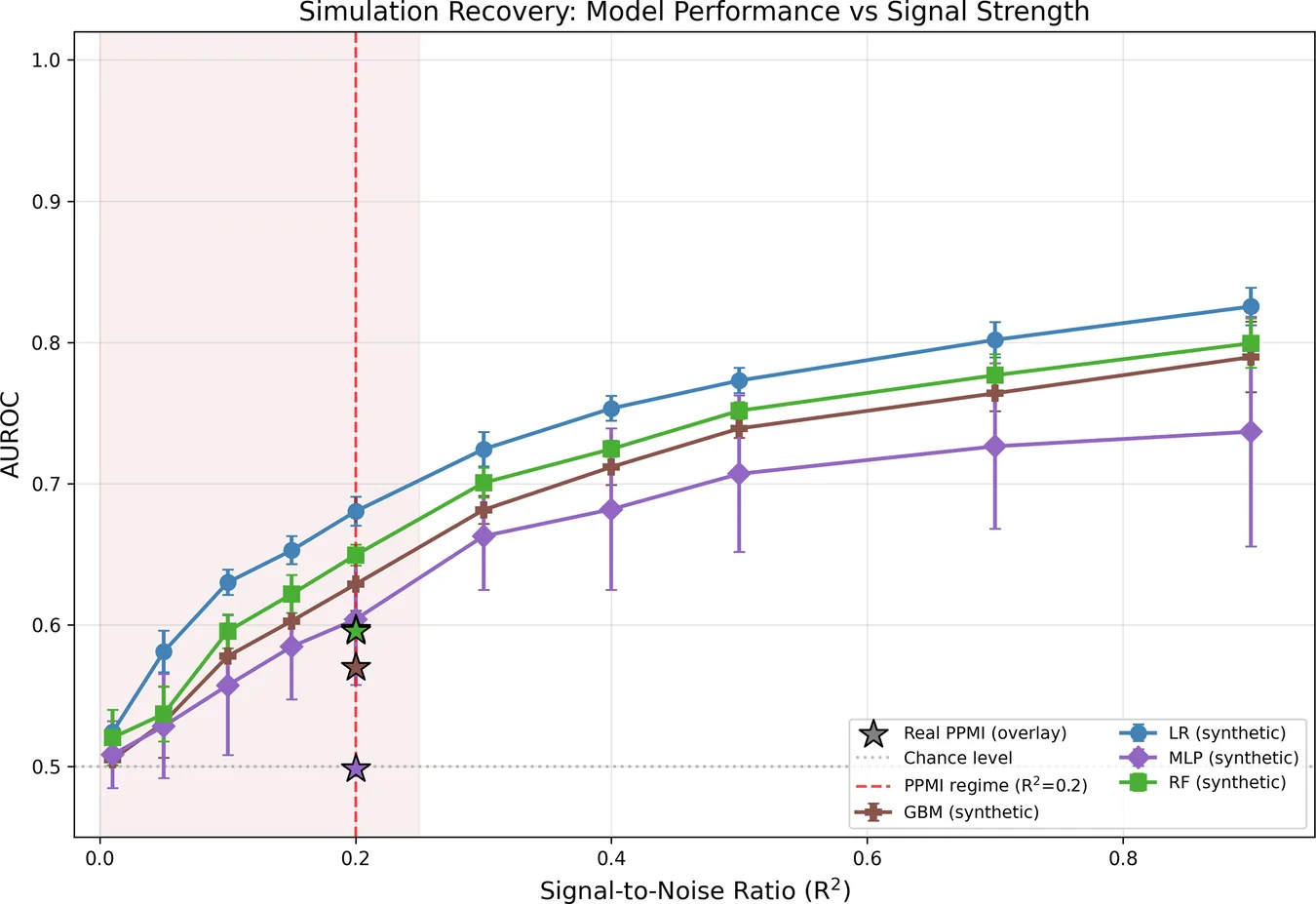
Simulation recovery: model performance vs signal strength. Synthetic data matching PPMI distributional parameters were generated at ten signal-to-noise levels. All models recover cognitive trajectories at high signal-to-noise (*R*² *≥* 0.50, AUROC > 0.70) but collapse uniformly at the signal levels present in PPMI cognitive data (R² approximately 0.20, red dashed line). Star markers indicate real PPMI performance, falling within the low signal-to-noise regime (*R*² approximately 0.10–0.15) of synthetic curves. The minimal inter-model spread at low SNR compared with the gain from increased signal strength confirms that the barrier is signal-limited.

To distinguish signal limitation from method limitation experimentally, we generated synthetic longitudinal data matching PPMI distributional parameters at ten signal-to-noise levels (*R*² from 0.01 to 0.90). Four representative models were evaluated at each level using identical five-fold cross-validation (Fig. 2).

At high signal-to-noise (*R*² *≥* 0.50), all models achieved AUROC exceeding 0.70, confirming that the analytical pipeline recovers cognitive trajectories when sufficient signal exists. At the signal level matching PPMI cognitive data (*R*² = 0.20), synthetic models converged to AUROC 0.60–0.68. Real PPMI data points fell within the synthetic curves at *R*² approximately 0.10–0.15, consistent with additional complexity in real data reducing effective signal below the nominal *R*².

The inter-model spread was minimal at low signal-to-noise (AUROC range approximately 0.08 at *R*² = 0.20) compared with the gain from increasing signal strength (AUROC gain approximately 0.15 from *R*² = 0.20 to *R*² = 0.50). Model selection contributed marginally to prediction performance relative to signal availability—formal evidence that the barrier is signal-limited (Fig. 2).

### Measurement fidelity determines the prediction ceiling

**Fig. 3:**
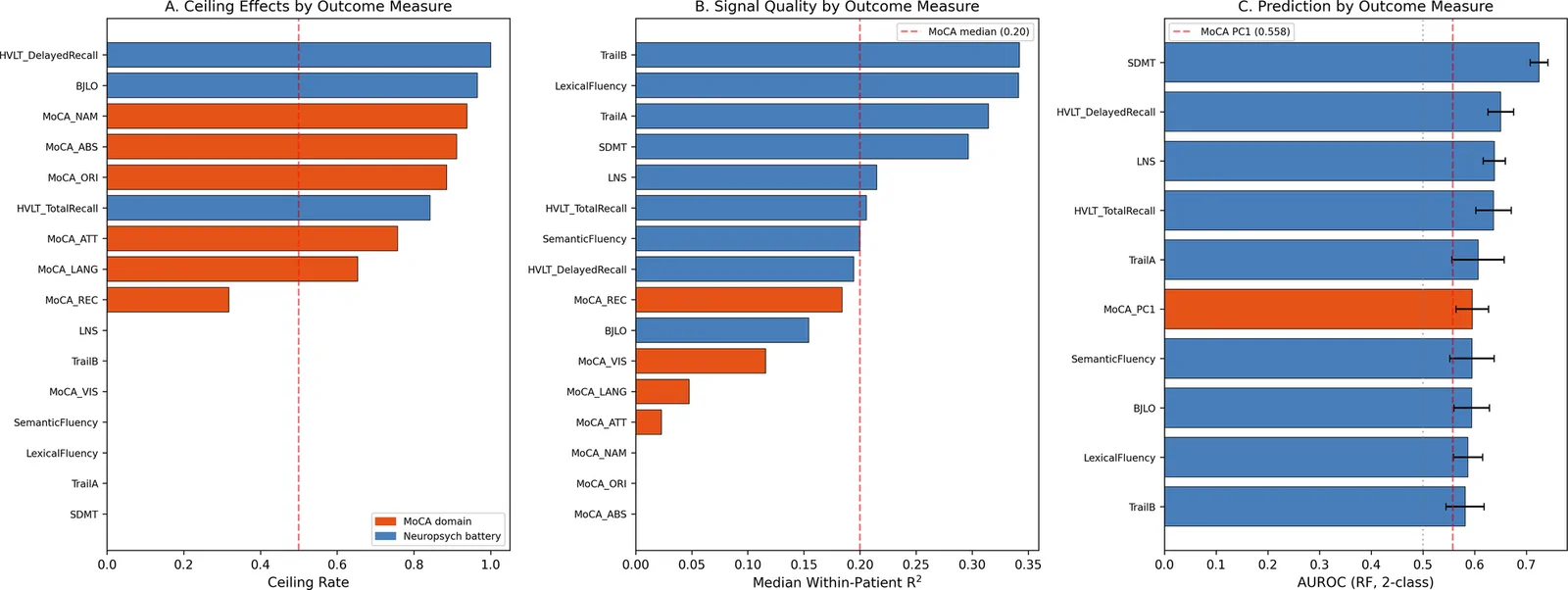
Measurement fidelity determines prediction performance (PPMI). **A** Ceiling effects by outcome measure. MoCA domains (orange) show severe ceiling effects (naming 94%, abstraction 91%, orientation 89%). Neuropsychological measures (blue) show no ceiling effects. **B** Within-patient *R*² by outcome measure. Measures with no ceiling effects show higher signal quality. **C** Prediction AUROC (random forest) by outcome measure. SDMT achieves 0.725, exceeding MoCA-based prediction (0.596) by 0.129 AUROC units.

If the prediction barrier reflects measurement fidelity rather than biological unpredictability, replacing coarse screening instruments with detailed neuropsychological tests should improve prediction. We tested this by substituting MoCA domain slopes with slopes derived from the PPMI neuropsychological battery—Symbol Digit Modalities Test (SDMT, processing speed), Hopkins Verbal Learning Test (HVLT, episodic memory), Letter-Number Sequencing (LNS, working memory), and Trail Making Tests (executive function/processing speed)—using identical baseline features and evaluation framework (Fig. 3).

The SDMT, which has zero ceiling effect and a 110-point dynamic range, achieved RF AUROC of 0.725 (SD 0.017)—exceeding MoCA-based prediction by 0.129 AUROC units (MoCA PC1: 0.596; Fig. 3C). HVLT delayed recall achieved 0.651 (SD 0.025), and LNS achieved 0.638 (SD 0.022). Across all measures, prediction performance correlated with signal quality: measures with lower ceiling rates and higher within-patient *R*² yielded higher AUROC (Fig. 3A–C). MoCA domains with severe ceiling effects (naming 94%, abstraction 91%, orientation 89%) showed near-zero within-patient *R*², while neuropsychological measures with no ceiling effects showed *R*² of 0.20–0.34.

These results establish measurement fidelity as a modifiable barrier: the same baseline features that fail to predict MoCA-derived trajectories achieve clinically meaningful discrimination when the outcome is measured with a higher-fidelity instrument.

### External validation in the NACC Parkinson disease cohort

To test whether the measurement fidelity finding generalizes beyond PPMI, we replicated the analysis in the NACC PD cohort (*N* = 523, median follow-up 3.9 years, mean age 72.2 years; Table 2). The NACC cohort differs from PPMI in enrollment criteria (clinic-based vs. research-enrolled), age (72 vs. 62 years), disease stage (variable vs. de novo), and available cognitive instruments.

**Table 2:** Cohort characteristics

Characteristic	PPMI (*N* = 1018)	NACC (*N* = 523)
Age, years, mean (SD)	62.1 (9.8)	72.2 (9.3)
Male, *n* (%)	672 (66.0)	357 (68.3)
Education, years, mean (SD)	15.5 (2.9)	16.2 (2.8)
Disease stage at enrollment	De novo	Variable
Median follow-up, years	4.2	3.9
Median visits per participant	8	5
Enrollment setting	Multicenter research	Clinic-based ADCs
Cognitive screening	MoCA (7 domains)	MMSE + MoCA
Neuropsychological battery	SDMT, HVLT, LNS, BJLO, TMT, fluency	LogMem, Craft Story, TMT, BNT, Digit Span, fluency

*ADC* Alzheimer’s Disease Center, *BJLO* Benton judgment of line orientation, *BNT* Boston naming test, *HVLT* Hopkins verbal learning test, *LNS* letter-number sequencing, *LogMem* logical memory, *MoCA* Montreal Cognitive Assessment, *MMSE* mini-mental state examination, *SDMT* symbol digit modalities test, *TMT* trail making test.

**Fig. 4:**
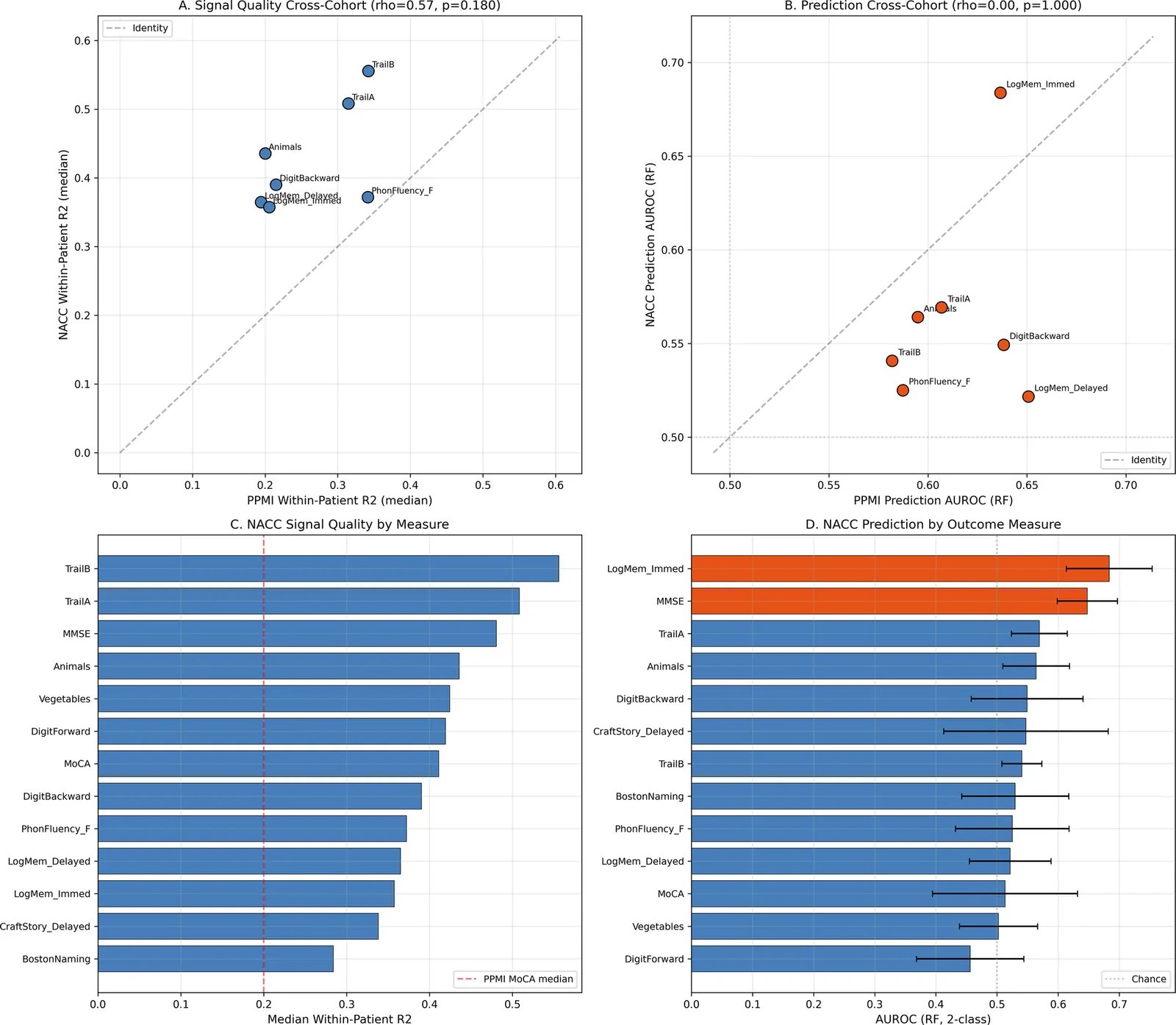
External validation in the NACC Parkinson disease cohort. **A** Cross-cohort concordance of within-patient R² across seven matched cognitive domains (Spearman ρ = 0.571). **B** Cross-cohort prediction AUROC comparison. **C** NACC signal quality by cognitive measure. **D** NACC prediction AUROC by outcome measure. Logical Memory Immediate (0.684) exceeds MMSE (0.648) and MoCA (0.513), replicating the PPMI measurement fidelity finding.

In NACC, Logical Memory Immediate achieved RF AUROC of 0.684 (SD 0.070), exceeding MMSE-based prediction (RF 0.648, SD 0.049) and MoCA-based prediction (RF 0.513, SD 0.119; Fig. 4D). Trail Making and semantic fluency showed intermediate performance (Fig. 4B, C). Cross-cohort signal quality patterns were concordant: the Spearman correlation between PPMI and NACC within-patient R² across seven matched cognitive domains was 0.571 (Fig. 4A). The cross-cohort AUROC ranking showed weaker concordance (*ρ* = 0.00; Fig. 4B), reflecting that the specific cognitive measures achieving the highest prediction differed between cohorts—SDMT in PPMI versus Logical Memory in NACC—while the overarching principle that detailed neuropsychological measures outperform screening instruments held in both.

The NACC replication confirms that the measurement fidelity barrier is not specific to PPMI’s de novo early-stage cohort but generalizes to a clinic-based population with more advanced disease.

### Signal characterization identifies the measurement barrier

**Fig. 5:**
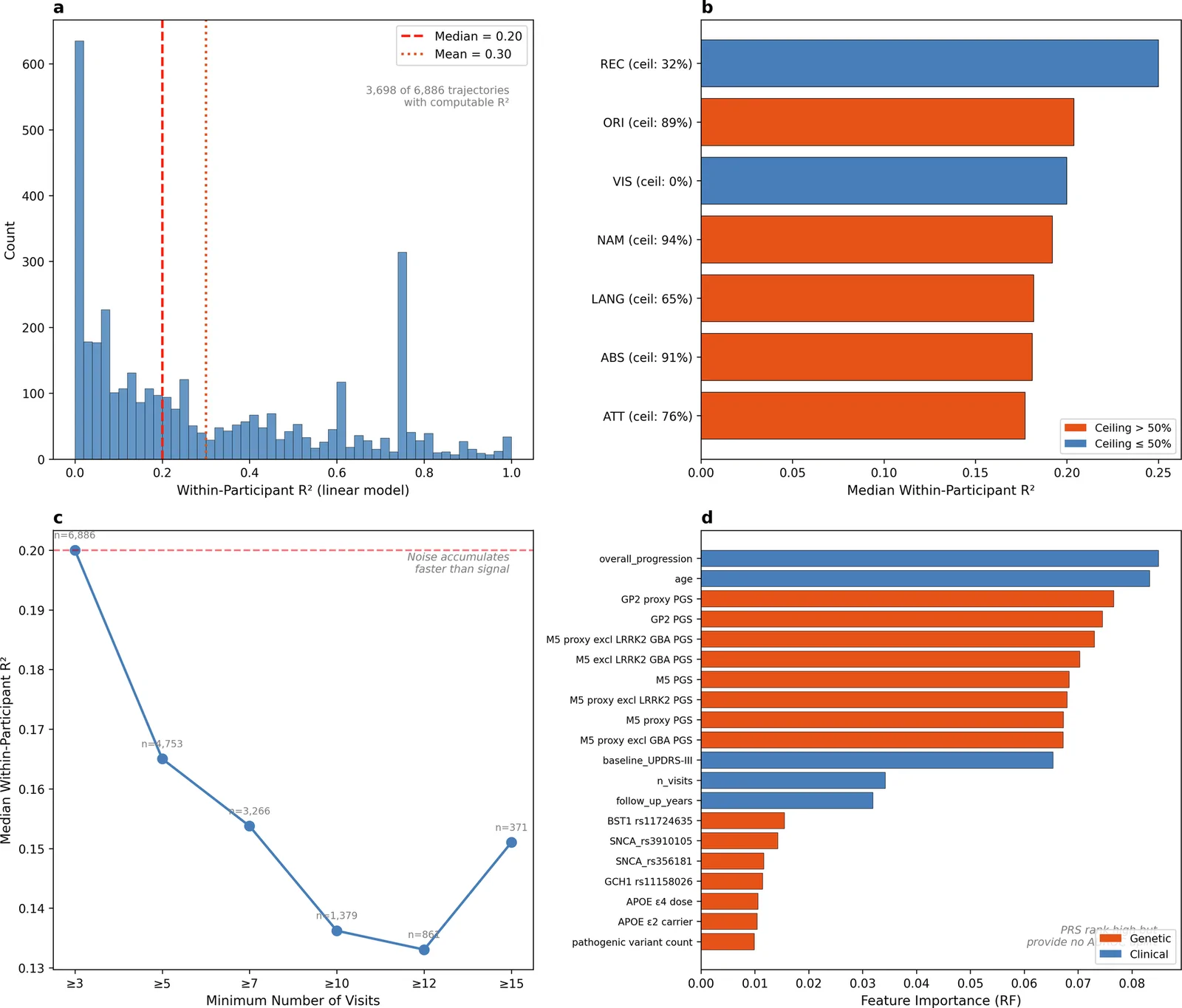
Signal characterization (PPMI). **a** Distribution of within-participant *R*² across 6886 participant-domain MoCA trajectories (3,698 with sufficient visits for *R*² estimation; median = 0.20, mean = 0.30). **b** *R*² by cognitive domain, ranked from lowest to highest, with ceiling rates annotated. Domains with severe ceiling effects (orange, >50%) show the lowest *R*². **c** Median *R*² decreases with increasing number of visits, indicating that additional observations accumulate noise at a rate exceeding signal clarification. **d** Top 20 feature importances for the cognitive target: polygenic risk scores (orange) rank among the top features but provide no AUROC improvement, indicating redundancy with clinical features (blue).

Within-patient *R*² is low. Across PPMI participant-domain trajectories, the median *R*² for a linear fit of MoCA domain score against time was 0.20 (mean 0.30; Fig. 5a). Only 15.3% of trajectories had *R*² exceeding 0.50. In NACC, within-patient *R*² was higher (median 0.28–0.56 depending on measure), reflecting longer follow-up and wider score ranges in a more advanced population.

The approximately 80% of score variance not captured by the linear temporal trend reflects multiple sources beyond measurement error: genuine biological variability (day-to-day cognitive fluctuations, medication state effects), non-linear progression patterns poorly captured by linear fits, practice effects from repeated test administration^13^, and transient state factors (fatigue, mood, testing conditions). The known psychometric limitations of MoCA for longitudinal monitoring^14,15^ further contribute to this composite variance. We therefore term this composite “non-signal variance” rather than “measurement noise.” Importantly, whether the low *R*² reflects measurement imprecision, biological state fluctuations, or non-linear dynamics, the practical implication is the same: the available cognitive screening data does not contain sufficient predictable linear signal for individual trajectory prediction, and higher-fidelity, higher-frequency assessment would address multiple components of this non-signal variance simultaneously.

Ceiling effects compress slope variance. Three of seven MoCA domains showed ceiling rates exceeding 85%: naming (estimated 94%), abstraction (89%), and orientation (88%). Neuropsychological tests without ceiling effects (SDMT, Trail Making, fluency) showed higher *R*² and higher prediction AUROC in both cohorts (Fig. 5b).

Longer follow-up does not improve MoCA signal. Median *R*² decreased with increasing visit count: from 0.20 at three visits to 0.13 at twelve or more visits (Fig. 5c), indicating that additional MoCA observations accumulate noise at a rate exceeding signal clarification.

Genetic features do not bridge the prediction gap. Adding 42 genetic variables including eight polygenic risk scores to a reduced five-clinical-variable set produced no significant cognitive improvement: RF AUROC changed from 0.558 to 0.559 (increase = 0.001, *p* = 0.937; Table 1). For the motor target, genetics provided a statistically significant improvement (LR: 0.840–0.862, *p* = 0.001). The eight polygenic risk scores ranked third through tenth in feature importance for the cognitive target yet provided no AUROC improvement, indicating redundancy with clinical features (Fig. 5d). APOE epsilon-4 carrier status showed no association with cognitive trajectory (Mann-Whitney *p* = 0.201, Cohen *d* = −0.08). GBA carrier status could not be evaluated as no carriers were present in the intersection cohort, a limitation reflecting PPMI enrollment of idiopathic PD participants; GBA is a major genetic risk factor for PD cognitive decline^16^ and future studies with GBA-enriched populations may reach different conclusions.

## Discussion

This multi-cohort eliminative study provides convergent evidence that individual-level cognitive trajectory prediction in PD is more strongly influenced by measurement fidelity than by model complexity. Three layers of convergent evidence support this conclusion: (1) systematic elimination of ten machine learning paradigm families across 26 configurations, with none exceeding clinically uninformative discrimination from MoCA-derived targets; (2) synthetic signal recovery experiments demonstrating that all models achieve strong performance at high signal-to-noise ratios but collapse uniformly at the levels present in clinical cognitive screening data; and (3) neuropsychological outcome substitution showing that higher-fidelity instruments improve prediction in both PPMI and NACC cohorts. These data suggest that the prediction ceiling is primarily set by the measurement instrument rather than by the algorithm, although domain-adapted algorithmic approaches remain an untested direction.

The asymmetry between motor subtype classification (AUROC = 0.869) and cognitive trajectory prediction (AUROC = 0.581) using identical features and analytical pipelines is methodologically decisive. If the features, preprocessing, cross-validation, or evaluation procedure were flawed, both targets would fail. The selective failure of the cognitive target establishes that the analytical approach is valid and localizes the barrier to the target-specific signal content.

The synthetic recovery experiment provides interventional evidence for the signal limitation claim. Rather than inferring from observational failure that signal is insufficient, we directly demonstrated that the same models achieve strong discrimination when provided adequate signal. The convergence of real PPMI performance with the low signal-to-noise regime of synthetic data confirms that the prediction ceiling is set by the data, not the algorithm. The minimal inter-model spread at low signal-to-noise (approximately 0.08 AUROC units) compared with the gain from signal improvement (approximately 0.15 units) quantifies the relative contributions: increasing signal strength matters approximately twice as much as optimal model selection.

The NACC replication extends the measurement fidelity finding from a de novo research cohort (PPMI) to a clinic-based population with more advanced disease. Despite differences in age (PPMI 62 years, NACC 72 years), enrollment criteria, and available cognitive instruments, the core finding held: detailed neuropsychological measures outperformed screening instruments for trajectory prediction. The cross-cohort *R*² correlation (*ρ* = 0.571) indicates concordant signal quality patterns across cohorts. This consistency suggests that the measurement barrier is a general property of current clinical cognitive assessment, not a cohort-specific artifact.

Our findings suggest that the path to individual-level cognitive trajectory prediction lies in measurement innovation rather than algorithmic refinement. In our experience managing individuals with PD across nearly two decades of functional neurosurgery practice, the difficulty of predicting individual cognitive trajectories has consistently limited shared decision-making for procedures such as deep brain stimulation, where cognitive reserve is a critical selection criterion. Our results provide direct evidence for two specific recommendations. First, replacing screening instruments with detailed neuropsychological batteries improved prediction by up to 0.129 AUROC units (PPMI SDMT), and this improvement generalized across cohorts. Second, measures without ceiling effects consistently outperformed ceiling-limited instruments, indicating that dynamic range is a key property of useful cognitive outcome measures. Additional strategies include higher-frequency cognitive assessment through digital biomarkers—such as smartphone-based active tapping tasks^17^, voice-based cognitive phenotyping, and passive gait monitoring via wearable accelerometers^18^—as well as ecological momentary assessment that captures within-day cognitive fluctuations^19^, longitudinal neuroimaging trajectories, and larger cohorts with richer phenotyping.

Several alternative explanations warrant consideration. Model ensembling was not evaluated; however, ensembling models that individually show weak discrimination has a theoretical ceiling constrained by individual model performance. Transfer learning from larger neurological cohorts represents a promising but untested direction. The sample size of 1018 (PPMI) and 523 (NACC) participants, while representative of the field, may be insufficient for methods requiring substantially larger training sets—though if a signal requires N substantially exceeding 1000 to detect, it is likely too weak for individual-level clinical prediction.

Model selection bias is a recognized concern when evaluating multiple algorithms on the same dataset, as the best-performing model’s estimate may be inflated by implicit multiple hypothesis testing^20^. However, our central conclusion rests on the uniform failure of all 26 configurations (all cognitive AUROC < 0.60), not on the selection of any single best model. Evaluating more models strengthens the negative conclusion by reducing the probability that an untested method would succeed. Furthermore, all hyperparameters were pre-specified without search, and the zero-shot TabPFN v2 foundation model provides an independent control. Structured strategies for handling limited datasets and class imbalance, as discussed by Trabassi et al.^20^, are relevant for positive-result studies but have limited bearing on a negative result where all methods converge to chance-level discrimination. More broadly, recent systematic reviews of clinical prediction model quality^21^ and reporting guidelines for AI-based prediction studies^22^ underscore the importance of transparent methodology, which we have addressed through pre-specified pipelines, fixed hyperparameters, and code availability.

Potential confounding structures may influence the observed prediction asymmetry. Treatment effects from dopaminergic medications may differentially affect motor and cognitive trajectories. Practice effects on repeated cognitive testing could inflate within-patient score variance without reflecting genuine cognitive change. Attrition bias may operate if participants with worse cognitive trajectories are more likely to withdraw, censoring the most informative cases. Our associational framework cannot fully disentangle these factors, and causal inference methods (e.g., instrumental variables, marginal structural models) applied to longitudinal PD data represent a complementary but untested direction.

An important limitation is that all ten paradigm families evaluated are general-purpose methods applied off-the-shelf to the PD cognitive prediction problem. Domain-adapted algorithms—such as architectures that explicitly model practice effects, attention-based models that learn test-retest reliability from individual trajectories, or multi-resolution models that fuse coarse screening with episodic detailed assessment—have not been explored and could potentially improve prediction. Motor subtype classification may be an inherently easier problem where baseline methods suffice, while cognitive trajectory prediction may require both measurement improvement and algorithmic innovation. The synthetic signal recovery analysis suggests that at the observed SNR level, the maximum gain from any model improvement is bounded (inter-model spread ~0.08 AUROC), but the interaction between measurement quality and domain-adapted innovation may be synergistic rather than additive.

Several limitations warrant consideration. First, the two cohorts analyzed — PPMI and NACC— are both North American and may not generalize to populations with different genetic backgrounds or healthcare systems. Second, GBA mutation carriers were absent from the PPMI analytic cohort, reflecting enrollment of idiopathic PD participants; GBA is a major genetic risk factor for PD cognitive decline^16^ and future studies with GBA-enriched populations may reach different conclusions. Third, the NACC PD sample (*N* = 523) is smaller than the PPMI sample, with MoCA coverage limited to 129 participants, reducing statistical power for some comparisons. Fourth, digital biomarkers and high-frequency monitoring data were not available, limiting evaluation to traditional clinical assessment modalities. Fifth, the clinical features available in the genetics comparison (GFS1) comprised five variables with complete data; the full 45-feature set had substantial missingness (up to 59% for DAT-SPECT), and imputation may have attenuated signals.

Sixth, the study does not evaluate decision-analytic utility, calibration under distributional shift, or integration within clinical workflows; these are necessary steps before the findings can inform clinical practice. Seventh, feature importances reflect predictive associations within the model, not causal relationships; bootstrap permutation importance analysis (200 resamples; Supplementary Fig. S1 and Supplementary Table S3) confirmed that only baseline MoCA total showed robust stability (top-10 inclusion 100%), while the overall ranking structure was not robust under perturbation (Spearman *ρ* = −0.034, *p* = 0.76). Eighth, prospective and temporally separated validation was not performed; all analyses are retrospective.

The two cohorts analyzed differ substantially in their data-generating processes, providing a natural test of transportability. PPMI enrolls de novo, untreated PD participants through research centers using standardized protocols, while NACC draws from clinic-based Alzheimer’s Disease Centers with heterogeneous referral patterns and broader disease severity. These differences introduce several forms of distributional shift: selection bias (earlier-stage, research-engaged participants in PPMI vs. later-stage, clinic-referred participants in NACC), covariate shift (mean age 62 vs. 72 years; de novo vs. variable disease stage), and measurement shift (MoCA domains vs. MMSE + MoCA; different neuropsychological batteries with partially overlapping cognitive domains). Quantitative covariate shift analysis on overlapping variables confirmed substantial differences: age (Cohen’s *d* = −0.935, KS *p* < 10⁻¹¹¹), baseline cognitive score (*d* = 0.959, *p* < 10⁻²⁰), education (*d* = 0.416), and sex composition (*d* = −0.214). A propensity score model achieved AUC = 0.715 ± 0.038 (combined *n* = 1827), indicating moderate but incomplete cohort separability (Supplementary Fig. S2).

The fact that the core finding—detailed neuropsychological measures outperform screening instruments for trajectory prediction—replicates across these markedly different data-generating processes provides evidence that the measurement fidelity barrier reflects a general property of current cognitive assessment instruments rather than dataset-specific correlations. However, both cohorts are North American, and the learned models were not applied across cohorts (i.e., training on PPMI and testing on NACC was not performed due to non-overlapping feature sets and cognitive instruments). Whether the measurement barrier generalizes to populations with different genetic backgrounds, healthcare systems, or cognitive assessment traditions remains an open empirical question. Future work should evaluate transportability using harmonized multinational cohorts and calibration metrics beyond discrimination.

This multi-cohort eliminative study provides convergent evidence that individual-level cognitive trajectory prediction in PD is more strongly constrained by measurement signal-to-noise ratio than by algorithmic sophistication, though the interaction between measurement quality and domain-adapted algorithmic innovation remains an open question. Across ten machine learning paradigm families, four feature modalities, and two independent cohorts, cognitive prediction from screening instruments remained clinically uninformative while higher-fidelity neuropsychological measures improved prediction in both populations. The within-patient *R*² of 0.20 for MoCA domain trajectories quantifies the non-signal variance barrier, reflecting the composite of measurement imprecision, biological state fluctuations, and non-linear dynamics. These findings suggest redirecting the research agenda from algorithm development toward measurement innovation: higher-frequency, higher-fidelity cognitive assessment instruments represent the most viable path toward enabling individual-level prediction. Prospective validation, evaluation of decision-analytic utility, and integration within clinical workflows are necessary before these findings inform clinical practice. The value of negative results for redirecting research effort has been underappreciated in biomedical science^23^.

## Methods

### Study populations

PPMI cohort. Data were obtained from the Parkinson’s Progression Markers Initiative (PPMI, www.ppmi-info.org), a multicenter longitudinal observational study. The PPMI protocol was approved by the respective Institutional Review Boards at each participating site; all participants provided written informed consent. We included 1018 participants with de novo PD who had at least three longitudinal visits with Montreal Cognitive Assessment (MoCA) domain scores and motor assessments. The median follow-up duration was 4.2 years (interquartile range 2.8–6.1 years), with a median of 8 visits per participant (range 3–18).

Motor subtypes were defined using data-driven clustering of longitudinal Unified Parkinson’s Disease Rating Scale Part III (UPDRS-III) trajectories, yielding four subtypes: Slow-Linear (*n* = 490), Accelerating (*n* = 288), Benign-Stable (*n* = 164), and Moderate-Steady (*n* = 74). Two participants classified as Fast-Aggressive were excluded from analyses requiring balanced class representation.

NACC cohort. Data were obtained from the National Alzheimer’s Coordinating Center (NACC) Uniform Data Set. We identified 1688 participants with a PD diagnosis (PARK = 1) and selected the 523 with three or more longitudinal visits. The NACC cohort differs from PPMI in enrollment criteria (clinic-based Alzheimer’s Disease Centers vs. multicenter research enrollment), age (mean 72.2 vs. 62.1 years), disease stage (variable vs. de novo), and available cognitive instruments (Table 2). The median follow-up was 3.9 years. NACC data collection was approved by participating Institutional Review Boards. These cohort differences provide a stringent test of generalizability.

#### Ethics statement

This study is a secondary analysis of de-identified data obtained from two publicly available research databases. The PPMI protocol was approved by the respective Institutional Review Boards at each participating site; all PPMI participants provided written informed consent prior to enrollment. NACC data collection was approved by participating Institutional Review Boards. Specific approval numbers are available upon request from the respective data coordinating centers. The present secondary analysis of fully de-identified data was determined to be exempt from additional IRB review in accordance with 45 CFR 46.104(d)(4).

### Feature modalities

The main cross-sectional and longitudinal benchmarks used all 45 available PPMI baseline features encompassing demographics, motor assessment, cognitive assessment, autonomic function, olfactory testing, cerebrospinal fluid biomarkers, and dopamine transporter imaging. All features are tabular—clinical rating scales, demographic variables, biomarker concentrations, and imaging-derived summary statistics—rather than raw imaging volumes or time-series waveforms. Key feature ranges: age (33–85 years), UPDRS-III total (0–132), MoCA total (0–30), CSF Aβ₁₋₄₂ (continuous, pg/mL), and DAT-SPECT caudate/putamen binding ratios (continuous).

To evaluate the contribution of genetic information under identical missingness conditions, an incremental feature comparison (Section 3.6) used a reduced set of five clinical variables with complete data across the genetics-clinical intersection cohort, denoted GFS1 through GFS4 (Supplementary Table 1). This design ensures that genetic uplift is measured without confounding by differential missingness.

#### GFS1 (Clinical)

Five baseline features with complete genetic overlap: age, baseline UPDRS-III total, overall motor progression rate, follow-up years, and number of visits.

#### GFS2 (+APOE)

GFS1 plus apolipoprotein E epsilon-4 allele carrier status, epsilon-4 dose (0, 1, or 2 copies), and epsilon-2 carrier status.

#### GFS3 (+Key Genes)

GFS2 plus glucocerebrosidase (GBA) carrier status, leucine-rich repeat kinase 2 (LRRK2) variant status, alpha-synuclein (SNCA) status, and total pathogenic variant count.

#### GFS4 (all genetics)

GFS3 plus eight polygenic risk scores (GP2 and META5 models with variant exclusions), whole-genome sequencing genotypes for six GBA variants (L444P, N370S, T408M, E365K, IVS2+1, 84GG), seven LRRK2 variants (including G2019S and R1441G/C), five SNCA variants, microtubule-associated protein tau (MAPT) rs17649553, and four additional risk loci (TMEM175, BST1, GCH1). Total: 47 features (5 clinical + 42 genetic).

For NACC prediction benchmarks, eight baseline features were used: age, sex, education, baseline MMSE, CDR global, CDR sum-of-boxes, GDS, and BMI.

### Cognitive outcome measures

MoCA domains (PPMI). Seven MoCA domain scores (visuospatial, naming, attention, language, abstraction, delayed recall, orientation) were extracted at each visit. Per-participant slopes were computed by ordinary least squares regression of domain score on visit time. The cognitive trajectory target was defined as a two-class split (fast vs. slow decline) based on the median of the first principal component of seven domain slopes.

Neuropsychological battery (PPMI). Nine neuropsychological tests from the PPMI battery were evaluated: Symbol Digit Modalities Test (SDMT, processing speed), Hopkins Verbal Learning Test total recall and delayed recall (HVLT, episodic memory), Letter-Number Sequencing (LNS, working memory), Benton Judgment of Line Orientation (BJLO, visuospatial), semantic fluency, lexical fluency, and Trail Making Tests A and B (processing speed, executive function). Each test’s cognitive domain, scoring range, ceiling rate, and fidelity advantage relative to MoCA are detailed in Supplementary Table S2. Per-participant slopes were computed identically to MoCA domains.

Neuropsychological battery (NACC). Thirteen cognitive measures were available: MMSE, MoCA, category fluency (animals, vegetables), Trail Making A and B, Boston Naming Test, Digit Span forward and backward, Logical Memory immediate and delayed, Craft Story delayed recall, and phonemic fluency. Missing data codes (−4, 8, 88, 95–99) were recoded as missing.

### Machine learning paradigm taxonomy

Ten paradigm families encompassing 26 model configurations were evaluated (Table 3). A summary of all prediction tasks, their definitions, evaluation setups, and cross-validation configurations is provided in Supplementary Table S1.

**Table 3:** Machine learning paradigm taxonomy

Category	Methods	Prediction type	N Configs
Cross-sectional	LR, RF, XGBoost, CatBoost, LightGBM, MLP, GBT, TabPFN v2	Classification	8
Survival	CoxPH, RSF, GBS	Time-to-event	3
Multi-task	SharedMLP, MMoE, PLE	Regression (7 domains)	3
Temporal	GRU, Latent ODE x2	Trajectory	3
Coupled dynamics	IndepLinODE, CoupledLinODE, CoupledNeuralODE	Trajectory	3
Causal/structural	NOTEARS, DAG-GNN, PC	Structure learning	3
Representation	VAE, beta-VAE, MC^2^VAE	Latent factors	3
Total			26

#### Cross-sectional classification (8 models)

Logistic regression (LR), random forest (RF, 500 trees), extreme gradient boosting (XGBoost), categorical boosting (CatBoost), light gradient boosting machine (LightGBM), multilayer perceptron (MLP, two hidden layers), gradient boosting tree (GBT), and TabPFN v2 (tabular prior-fitted network, a foundation model pre-trained on synthetic tabular tasks^3^).

#### Survival analysis (3 models)

Cox proportional hazards (CoxPH), random survival forest (RSF), and gradient boosting survival (GBS), each evaluated with split-conformal prediction intervals^24^.

#### Multi-task learning (3 models)

Shared multilayer perceptron (SharedMLP), multi-gate mixture-of-experts (MMoE^5^), and progressive layered extraction (PLE^4^).

#### Temporal modeling (3 models)

Gated recurrent unit (GRU), latent ordinary differential equation (Latent ODE^6^), and a large-capacity Latent ODE variant.

#### Coupled dynamics (3 models)

Independent linear ODE (IndepLinODE), coupled linear ODE (CoupledLinODE), and coupled neural ODE (CoupledNeuralODE).

#### Causal and structural learning (3 models)

NOTEARS^8^, DAG-GNN^7^, and the PC algorithm^25^.

#### Representation learning (3 models)

Standard variational autoencoder (VAE), beta-VAE, and multi-channel causal VAE (MC^2^VAE).

### Synthetic signal recovery design

To distinguish signal limitation from method limitation experimentally, we generated synthetic longitudinal data matching PPMI distributional parameters (means, standard deviations, and correlation structure) at ten signal-to-noise levels (*R*² from 0.01 to 0.90). At each level, a sparse true signal (10% of features informative) was embedded in correlated Gaussian noise. The signal-to-noise ratio was controlled by adjusting noise variance relative to signal variance. Four representative models—logistic regression, random forest, gradient boosting, and multilayer perceptron—were evaluated at each level using five-fold cross-validation with five independent repetitions per level. Real PPMI cognitive prediction performance was overlaid on the synthetic curves to identify the effective signal-to-noise regime.

Specifically, features X ∈ ℝN×p were sampled from a multivariate Gaussian distribution N(μ̂, Σ̂) where μ̂ and Σ̂ were estimated from the PPMI baseline feature matrix. The cognitive outcome was generated as y_latent = Xβ + ε, where β ∈ ℝp was sparse with ceil(0.1p) non-zero entries drawn independently from N(0, 1) and ε ~ N(0, *σ*²_noise). The noise variance was set to *σ*²_noise = Var(Xβ) × (1 − SNR) / SNR to achieve the target signal-to-noise ratio, defined as SNR = Var(Xβ) / [Var(Xβ) + *σ*²_noise]. The binary classification target was y = 1(y_latent > median(y_latent)). Five independent datasets were generated at each of ten SNR levels (0.01, 0.05, 0.10, 0.15, 0.20, 0.30, 0.40, 0.50, 0.70, 0.90) to estimate performance variance.

### Signal characterization

Four signal properties were quantified: (1) within-patient *R*² from linear regression of MoCA domain score on visit time; (2) ceiling effects (percentage of observations at maximum possible score per domain); (3) feature-target mutual information via random forest importances; and (4) linear vs. quadratic model comparison via AIC and F-test.

A ceiling effect occurs when a measurement instrument’s maximum possible score is reached by a large proportion of individuals, compressing the observable range and preventing discrimination among those at or near the top of the ability distribution. For example, the MoCA naming subscore (range 0–3) shows a ceiling rate of 94%: nearly all PD participants score 3/3 at every visit, producing near-zero variance in longitudinal slopes and rendering the measure uninformative for trajectory prediction. By contrast, the SDMT (range 0–110) shows a ceiling rate of 0%, distributing observations across the full range and preserving slope variance.

### Evaluation framework

All classification tasks used five-fold stratified cross-validation with a fixed random seed (42). All models used pre-specified hyperparameters without data-driven tuning. No hyperparameter search was conducted, eliminating the risk of information leakage. Critically, no data-driven feature selection was performed: all 45 available PPMI features were used for the main benchmarks, and the feature set was fixed before any model evaluation. The full modeling pipeline—including preprocessing, feature set definition, and model configurations—was specified prior to evaluation, ensuring that no information from the test folds influenced any upstream step. The NACC cohort (*N* = 523) serves as a fully independent external validation cohort, not an internal data split: it differs from PPMI in enrollment criteria, age distribution, disease stage, and available cognitive instruments. The zero-shot foundation model TabPFN v2 achieved comparable cognitive prediction performance (AUROC 0.581), indicating that hyperparameter tuning is unlikely to meaningfully alter the conclusions.

Primary metrics were AUROC, macro-averaged F1 score, and balanced accuracy. Regression tasks reported mean absolute error, root mean squared error, and Spearman's rank correlation. All performance metrics are reported as mean and standard deviation across five folds. Pairwise comparisons used paired t-tests across folds with a significance threshold of alpha = 0.05.

### Software and reproducibility

Analyses were conducted in Python 3.11 using scikit-learn 1.5, PyTorch 2.1, torchdiffeq 0.2.5, causal-learn 0.1.4, and TabPFN 2.0. Computations were performed on an NVIDIA RTX 2000 Ada GPU. Code is available at [repository URL to be added upon acceptance].

A private repository link for reviewer access has been provided in the cover letter accompanying this revision. The complete analysis code, including all model configurations, preprocessing pipelines, and evaluation scripts, will be deposited in a public GitHub repository upon acceptance.

## Supplementary information

Supplementary_Figure_S1

Supplementary_Figure_S2

Supplementary_Table1

Supplementary_TableS2

Supplementary_TableS3

Figures legend

## Data Availability

The data used in this study were obtained from two sources. PPMI data were obtained from the Parkinson’s Progression Markers Initiative database (www.ppmi-info.org). PPMI is funded by the Michael J. Fox Foundation for Parkinson’s Research and funding partners, including AbbVie, Allergan, Amathus Therapeutics, Avid Radiopharmaceuticals, Biogen, BioLegend, Bristol-Myers Squibb, Celgene, Denali, GE Healthcare, Genentech, GlaxoSmithKline, Golub Capital, Handl Therapeutics, Insitro, Janssen Neuroscience, Lilly, Lundbeck, Merck, Meso Scale Diagnostics, Pfizer, Piramal, Prevail Therapeutics, Roche, Sanofi Genzyme, Servier, Takeda, Teva, UCB, Verily, and Voyager Therapeutics. NACC data were obtained from the National Alzheimer’s Coordinating Center (NACC) database, funded by NIA/NIH grant U24 AG072122.

## Code availability

A private repository link for reviewer access has been provided in the cover letter. The complete analysis code, including all model configurations, preprocessing pipelines, and evaluation scripts, will be deposited in a public GitHub repository upon acceptance.
