# Supplementary material for: Measurement fidelity, not model complexity, governs individual cognitive trajectory prediction in Parkinson's disease
Source: NPJ Digit Med. 2026 Jun 6;9:698. doi: 10.1038/s41746-026-02681-8 (PMC13572477; doi:10.1038/s41746-026-02681-8)
Supplement: Supplementary file 1 [file 41746_2026_2681_MOESM3_ESM.pdf]

Supplementary Table 1. Genetics Feature Set (GFS) Composition for Incremental Feature Comparison

Feature Set	Variables Included	Description
GFS1 (Clinical)	Age, baseline UPDRS-III total, overall motor progression rate, follow-up years, number of visits	Five baseline clinical features with complete data across the genetics-clinical intersection cohort
GFS2 (+APOE)	GFS1 + APOE ε4 carrier status, ε4 dose (0/1/2 copies), ε2 carrier status	Clinical features augmented with apolipoprotein E allele information
GFS3 (+Key Genes)	GFS2 + GBA carrier status, LRRK2 variant status, SNCA status, total pathogenic variant count	Addition of established PD-associated genetic variants
GFS4 (All Genetics)	GFS3 + 8 polygenic risk scores (GP2 and META5 models), 6 GBA variant genotypes (L444P, N370S, T408M, E365K, IVS2+1, 84GG), total coding variant burden	Full genetic feature set including polygenic risk and individual variant genotypes

APOE, apolipoprotein E; GBA, glucocerebrosidase; LRRK2, leucine-rich repeat kinase 2; SNCA, alpha-synuclein; UPDRS-III, Unified Parkinson's Disease Rating Scale Part III. The incremental design ensures that genetic uplift is measured without confounding by differential missingness.
