# Supplementary material for: Measurement fidelity, not model complexity, governs individual cognitive trajectory prediction in Parkinson's disease
Source: NPJ Digit Med. 2026 Jun 6;9:698. doi: 10.1038/s41746-026-02681-8 (PMC13572477; doi:10.1038/s41746-026-02681-8)
Supplement: Supplementary file 2 [file 41746_2026_2681_MOESM4_ESM.docx]

**Supplementary Table S2. Cognitive instrument fidelity across PPMI discovery and NACC replication cohorts.**

Per-instrument cognitive domain, scoring range, ceiling rate, Random Forest trajectory-prediction AUROC, and Δ AUROC relative to the cohort-matched MoCA reference. PPMI discovery section (n = 1,018 with ≥3 longitudinal MoCA visits) uses MoCA PC1 (first principal component of the seven MoCA domain slopes) as the delta reference; NACC replication uses MoCA total score as the delta reference. Higher-fidelity instruments (zero or near-zero ceiling, wider dynamic range) consistently outperform MoCA-derived targets in both cohorts; SDMT in PPMI (Δ = +0.129) and Logical Memory Immediate in NACC (Δ = +0.171) constitute the principal evidence in Figures 3 and 4 of the main text. AUROC values reflect 5-fold cross-validation on baseline imaging + clinical features.

| **Instrument** | **Cohort** | **Cognitive Domain** | **Scoring Range** | **Ceiling Rate** | **RF AUROC (mean ± SD)** | **Δ AUROC vs MoCA** | **n (patients)** |
| --- | --- | --- | --- | --- | --- | --- | --- |
| SDMT | PPMI | Processing Speed | 0–110 | 0% | 0.725 ± 0.017 | +0.129 | 979 |
| HVLT-R Total Recall | PPMI | Episodic Memory | 0–36 | 84% | 0.637 ± 0.034 | +0.041 | 980 |
| HVLT-R Delayed Recall | PPMI | Episodic Memory | 0–12 | 100% | 0.651 ± 0.025 | +0.055 | 978 |
| Letter–Number Sequencing | PPMI | Working Memory | 0–21 | <1% | 0.638 ± 0.021 | +0.043 | 979 |
| Benton Judgment of Line Orientation | PPMI | Visuospatial | 0–15 | 97% | 0.595 ± 0.034 | −0.001 | 979 |
| Semantic Fluency (Animals) | PPMI | Semantic Fluency | open count | 0% | 0.595 ± 0.043 | −0.001 | 980 |
| Lexical Fluency | PPMI | Phonemic Fluency | open count | 0% | 0.587 ± 0.028 | −0.008 | 735 |
| Trail Making A | PPMI | Processing Speed | open (seconds) | 0% | 0.607 ± 0.051 | +0.011 | 747 |
| Trail Making B | PPMI | Executive Function | open (seconds) | 0% | 0.582 ± 0.037 | −0.014 | 723 |
| MoCA PC1 (composite) | PPMI | MoCA Composite | PC1 of 7 domains | — | 0.596 ± 0.032 | (reference) | 1015 |
| MoCA Visuospatial | PPMI | Visuospatial | 0–5 | 0% | (domain only) | (reference) | 982 |
| MoCA Naming | PPMI | Language (naming) | 0–3 | 94% | (domain only) | (reference) | 982 |
| MoCA Attention | PPMI | Attention | 0–6 | 76% | (domain only) | (reference) | 982 |
| MoCA Language | PPMI | Language | 0–3 | 65% | (domain only) | (reference) | 982 |
| MoCA Abstraction | PPMI | Abstraction | 0–2 | 91% | (domain only) | (reference) | 982 |
| MoCA Delayed Recall | PPMI | Episodic Memory | 0–5 | 32% | (domain only) | (reference) | 982 |
| MoCA Orientation | PPMI | Orientation | 0–6 | 89% | (domain only) | (reference) | 982 |
| Logical Memory – Immediate | NACC | Episodic Memory | 0–25 | 0% | 0.684 ± 0.070 | +0.171 | 234 |
| Logical Memory – Delayed | NACC | Episodic Memory | 0–25 | 17% | 0.522 ± 0.067 | +0.009 | 255 |
| Craft Story – Delayed | NACC | Episodic Memory | open (paraphrase) | 0% | 0.547 ± 0.135 | +0.034 | 140 |
| MMSE | NACC | Global Cognition | 0–30 | 24% | 0.648 ± 0.049 | +0.135 | 272 |
| Boston Naming Test | NACC | Language | open | 19% | 0.530 ± 0.088 | +0.017 | 260 |
| Animals (Semantic Fluency) | NACC | Semantic Fluency | open count | 0% | 0.564 ± 0.055 | +0.051 | 386 |
| Vegetables (Semantic Fluency) | NACC | Semantic Fluency | open count | 0% | 0.502 ± 0.064 | −0.011 | 330 |
| Phonemic Fluency – F | NACC | Phonemic Fluency | open count | 0% | 0.525 ± 0.093 | +0.012 | 136 |
| Trail Making A (NACC) | NACC | Processing Speed | open (seconds) | 0% | 0.569 ± 0.046 | +0.056 | 371 |
| Trail Making B (NACC) | NACC | Executive Function | open (seconds) | 0% | 0.541 ± 0.033 | +0.028 | 323 |
| Digit Forward | NACC | Attention | open (digits) | 2% | 0.456 ± 0.088 | −0.057 | 80 |
| Digit Backward | NACC | Working Memory | open (digits) | <1% | 0.549 ± 0.092 | +0.036 | 108 |
| MoCA (NACC) | NACC | Global Cognition | 0–30 | 7% | 0.513 ± 0.119 | (reference) | 129 |

*Abbreviations: AUROC, area under the receiver operating characteristic curve; BJLO, Benton Judgment of Line Orientation; HVLT-R, Hopkins Verbal Learning Test – Revised; LNS, Letter–Number Sequencing; MMSE, Mini-Mental State Examination; MoCA, Montreal Cognitive Assessment; NACC, National Alzheimer's Coordinating Center; PC1, first principal component; PPMI, Parkinson's Progression Markers Initiative; RF, Random Forest; SD, standard deviation; SDMT, Symbol Digit Modalities Test. "Domain only" indicates the MoCA subscore was used for ceiling-rate analysis (Figure 5a) and within-patient R² estimation; standalone trajectory AUROC was not computed for individual MoCA subscores. Δ AUROC for MoCA subscores is therefore listed as "(reference)" because the comparison framework substitutes whole-instrument outcomes (SDMT, HVLT, etc.) in place of the MoCA composite reference (PPMI: MoCA PC1; NACC: MoCA total).*
