# Supplementary material for: Measurement fidelity, not model complexity, governs individual cognitive trajectory prediction in Parkinson's disease
Source: NPJ Digit Med. 2026 Jun 6;9:698. doi: 10.1038/s41746-026-02681-8 (PMC13572477; doi:10.1038/s41746-026-02681-8)
Supplement: Supplementary file 3 [file 41746_2026_2681_MOESM5_ESM.docx]

**Supplementary Table S3. Bootstrap permutation feature-importance stability ranking for the cognitive trajectory target (Random Forest, 200 bootstrap resamples; PPMI, n = 1,018).**

Features ordered by bootstrap-aggregated mean permutation importance (descending). Top-10 / Top-20 stability is the empirical frequency that the feature appears in the top-10 / top-20 of the original-fit importance ranking across the 200 bootstrap resamples (100% = appears in every resample). Rank Shift = Original Rank − Bootstrap Rank; positive values indicate features promoted by bootstrap aggregation. baseline_moca_total is the only feature with Top-10 stability of 100% (rank shift +21), matching the manuscript text statement that "only baseline MoCA total showed robust stability (top-10 inclusion 100%)." Spearman rank correlation between the bootstrap-aggregated and the original single-fit importance profiles is rho = -0.034 (p = 0.76; n = 81 features), indicating that the overall ranking structure is not robust under perturbation; this analysis underpins the seventh limitation in the Discussion. See Supplementary Figure S1 for graphical display of the top-ranked features.

| **Bootstrap Rank** | **Feature** | **Bootstrap Mean Importance** | **95% CI** | **Top-10 Stability** | **Top-20 Stability** | **Original Importance** | **Original Rank** | **Rank Shift** |
| --- | --- | --- | --- | --- | --- | --- | --- | --- |
| 1 | baseline_moca_total | +0.0253 | [+0.0136, +0.0397] | 100% | 100% | +0.0247 | 22 | +21 |
| 2 | Occipital_L_Thickness | +0.0084 | [-0.0022, +0.0205] | 85% | 91% | +0.0338 | 2 | +0 |
| 3 | Temporal_L_Thickness | +0.0055 | [-0.0056, +0.0171] | 73% | 81% | +0.0323 | 4 | +1 |
| 4 | Caudate_SBR_LR_Ratio | +0.0036 | [-0.0024, +0.0098] | 67% | 82% | +0.0146 | 25 | +21 |
| 5 | Caudate_SBR_Asymmetry | +0.0035 | [-0.0024, +0.0093] | 63% | 75% | +0.0144 | 26 | +21 |
| 6 | Cingulate_L_Thickness | +0.0030 | [-0.0067, +0.0132] | 54% | 66% | +0.0323 | 5 | -1 |
| 7 | Occipital_Thickness | +0.0013 | [-0.0064, +0.0096] | 38% | 54% | +0.0301 | 6 | -1 |
| 8 | NP3PTRMR | +0.0012 | [-0.0012, +0.0041] | 16% | 57% | +0.0043 | 58 | +50 |
| 9 | ICV | +0.0011 | [-0.0105, +0.0114] | 37% | 53% | +0.0340 | 1 | -8 |
| 10 | Temporal_Thickness | +0.0010 | [-0.0064, +0.0079] | 34% | 52% | +0.0278 | 12 | +2 |
| 11 | Striatum_SBR_LR_Ratio | +0.0009 | [-0.0040, +0.0053] | 22% | 49% | +0.0101 | 32 | +21 |
| 12 | scopa_aut_total | +0.0008 | [-0.0071, +0.0090] | 36% | 52% | +0.0279 | 10 | -2 |
| 13 | NP3LGAGL | +0.0008 | [-0.0024, +0.0043] | 14% | 42% | +0.0050 | 52 | +39 |
| 14 | Striatum_SBR_Asymmetry | +0.0008 | [-0.0031, +0.0047] | 20% | 45% | +0.0108 | 27 | +13 |
| 15 | NP3BRADY | +0.0006 | [-0.0023, +0.0037] | 9% | 40% | +0.0049 | 54 | +39 |
| 16 | Caudate_SBR_L | +0.0006 | [-0.0048, +0.0051] | 21% | 45% | +0.0106 | 29 | +13 |
| 17 | Temporal_R_Thickness | +0.0006 | [-0.0076, +0.0076] | 30% | 48% | +0.0278 | 11 | -6 |
| 18 | upsit_total | +0.0004 | [-0.0023, +0.0025] | 6% | 30% | +0.0051 | 49 | +31 |
| 19 | NP3PSTBL | +0.0003 | [-0.0018, +0.0026] | 4% | 23% | +0.0028 | 75 | +56 |
| 20 | Striatum_SBR_R | +0.0003 | [-0.0028, +0.0038] | 12% | 32% | +0.0095 | 35 | +15 |
| 21 | education_years | +0.0003 | [-0.0043, +0.0034] | 11% | 36% | +0.0096 | 33 | +12 |
| 22 | NP3FTAPR | +0.0003 | [-0.0025, +0.0033] | 10% | 28% | +0.0051 | 51 | +29 |
| 23 | Caudate_SBR | +0.0003 | [-0.0037, +0.0035] | 12% | 40% | +0.0095 | 34 | +11 |
| 24 | NP3PRSPL | +0.0003 | [-0.0031, +0.0032] | 4% | 28% | +0.0052 | 45 | +21 |
| 25 | NP3RIGLL | +0.0002 | [-0.0039, +0.0035] | 6% | 28% | +0.0039 | 62 | +37 |
| 26 | Caudate_SBR_R | +0.0002 | [-0.0036, +0.0037] | 10% | 32% | +0.0092 | 36 | +10 |
| 27 | NP3RIGLU | +0.0001 | [-0.0024, +0.0019] | 2% | 20% | +0.0041 | 61 | +34 |
| 28 | NP3HMOVR | +0.0001 | [-0.0048, +0.0038] | 9% | 29% | +0.0051 | 50 | +22 |
| 29 | NP3FRZGT | +0.0000 | [-0.0001, +0.0001] | 0% | 0% | +0.0002 | 80 | +51 |
| 30 | baseline_ledd | +0.0000 | [+0.0000, +0.0000] | 0% | 0% | +0.0000 | 81 | +51 |
| 31 | NP3RTARU | -0.0000 | [-0.0021, +0.0019] | 2% | 14% | +0.0041 | 59 | +28 |
| 32 | NP3RTALU | -0.0001 | [-0.0015, +0.0012] | 0% | 6% | +0.0028 | 74 | +42 |
| 33 | NP3LGAGR | -0.0001 | [-0.0027, +0.0023] | 2% | 16% | +0.0036 | 66 | +33 |
| 34 | NP3RTALJ | -0.0002 | [-0.0009, +0.0005] | 0% | 2% | +0.0012 | 78 | +44 |
| 35 | NP3RIGRU | -0.0002 | [-0.0029, +0.0021] | 3% | 18% | +0.0052 | 46 | +11 |
| 36 | NP3RTALL | -0.0002 | [-0.0013, +0.0004] | 0% | 0% | +0.0011 | 79 | +43 |
| 37 | NP3FACXP | -0.0002 | [-0.0032, +0.0022] | 2% | 16% | +0.0051 | 48 | +11 |
| 38 | NP3RTARL | -0.0003 | [-0.0017, +0.0007] | 0% | 4% | +0.0017 | 77 | +39 |
| 39 | NHY | -0.0003 | [-0.0027, +0.0017] | 1% | 8% | +0.0032 | 70 | +31 |
| 40 | NP3GAIT | -0.0003 | [-0.0020, +0.0013] | 1% | 6% | +0.0034 | 68 | +28 |
| 41 | Striatum_SBR_L | -0.0003 | [-0.0041, +0.0026] | 4% | 16% | +0.0090 | 38 | -3 |
| 42 | MSEADLG | -0.0003 | [-0.0028, +0.0021] | 0% | 10% | +0.0052 | 47 | +5 |
| 43 | NP3FTAPL | -0.0004 | [-0.0032, +0.0017] | 0% | 10% | +0.0043 | 57 | +14 |
| 44 | age | -0.0004 | [-0.0098, +0.0076] | 25% | 39% | +0.0330 | 3 | -41 |
| 45 | NP3RISNG | -0.0004 | [-0.0022, +0.0008] | 0% | 2% | +0.0026 | 76 | +31 |
| 46 | NP3POSTR | -0.0004 | [-0.0033, +0.0018] | 2% | 10% | +0.0041 | 60 | +14 |
| 47 | NP3TTAPR | -0.0004 | [-0.0033, +0.0015] | 1% | 6% | +0.0044 | 56 | +9 |
| 48 | NP3PTRML | -0.0004 | [-0.0023, +0.0011] | 0% | 6% | +0.0030 | 73 | +25 |
| 49 | NP3RTCON | -0.0005 | [-0.0032, +0.0019] | 0% | 14% | +0.0049 | 55 | +6 |
| 50 | NP3HMOVL | -0.0005 | [-0.0042, +0.0017] | 1% | 10% | +0.0049 | 53 | +3 |
| 51 | sex | -0.0005 | [-0.0039, +0.0021] | 2% | 12% | +0.0038 | 63 | +12 |
| 52 | NP3KTRMR | -0.0005 | [-0.0023, +0.0013] | 1% | 6% | +0.0037 | 65 | +13 |
| 53 | NP3RIGRL | -0.0005 | [-0.0028, +0.0016] | 0% | 5% | +0.0030 | 72 | +19 |
| 54 | Putamen_SBR_Asymmetry | -0.0006 | [-0.0053, +0.0030] | 8% | 24% | +0.0103 | 30 | -24 |
| 55 | NP3KTRML | -0.0006 | [-0.0027, +0.0013] | 0% | 4% | +0.0035 | 67 | +12 |
| 56 | Putamen_SBR_LR_Ratio | -0.0006 | [-0.0053, +0.0040] | 8% | 24% | +0.0103 | 31 | -25 |
| 57 | baseline_pigd | -0.0006 | [-0.0041, +0.0021] | 3% | 16% | +0.0070 | 43 | -14 |
| 58 | NP3TTAPL | -0.0006 | [-0.0034, +0.0020] | 1% | 6% | +0.0053 | 44 | -14 |
| 59 | Putamen_SBR_R | -0.0006 | [-0.0040, +0.0022] | 2% | 16% | +0.0090 | 37 | -22 |
| 60 | baseline_tremor | -0.0006 | [-0.0046, +0.0024] | 3% | 16% | +0.0081 | 41 | -19 |
| 61 | NP3RIGN | -0.0007 | [-0.0038, +0.0012] | 0% | 6% | +0.0037 | 64 | +3 |
| 62 | NP3SPCH | -0.0007 | [-0.0038, +0.0009] | 0% | 0% | +0.0030 | 71 | +9 |
| 63 | NP3PRSPR | -0.0007 | [-0.0025, +0.0009] | 0% | 3% | +0.0034 | 69 | +6 |
| 64 | Striatum_SBR | -0.0007 | [-0.0034, +0.0019] | 0% | 12% | +0.0083 | 40 | -24 |
| 65 | tremor_pigd_ratio | -0.0009 | [-0.0072, +0.0032] | 6% | 20% | +0.0107 | 28 | -37 |
| 66 | Cingulate_Thickness | -0.0009 | [-0.0092, +0.0060] | 16% | 32% | +0.0300 | 7 | -59 |
| 67 | Putamen_SBR | -0.0010 | [-0.0037, +0.0015] | 1% | 8% | +0.0078 | 42 | -25 |
| 68 | Putamen_SBR_L | -0.0010 | [-0.0041, +0.0017] | 2% | 5% | +0.0086 | 39 | -29 |
| 69 | CorticalThickness_Mean | -0.0011 | [-0.0083, +0.0055] | 18% | 30% | +0.0266 | 19 | -50 |
| 70 | baseline_updrs3 | -0.0011 | [-0.0060, +0.0044] | 8% | 22% | +0.0184 | 24 | -46 |
| 71 | Occipital_R_Thickness | -0.0013 | [-0.0097, +0.0060] | 17% | 28% | +0.0290 | 8 | -63 |
| 72 | Parietal_R_Thickness | -0.0021 | [-0.0118, +0.0058] | 12% | 22% | +0.0271 | 14 | -58 |
| 73 | Frontal_R_Thickness | -0.0021 | [-0.0114, +0.0046] | 11% | 22% | +0.0274 | 13 | -60 |
| 74 | Parietal_L_Thickness | -0.0026 | [-0.0102, +0.0036] | 7% | 16% | +0.0285 | 9 | -65 |
| 75 | Motor_R_Thickness | -0.0029 | [-0.0108, +0.0030] | 4% | 10% | +0.0267 | 17 | -58 |
| 76 | Frontal_Thickness | -0.0031 | [-0.0102, +0.0035] | 4% | 10% | +0.0240 | 23 | -53 |
| 77 | Frontal_L_Thickness | -0.0034 | [-0.0114, +0.0039] | 6% | 13% | +0.0257 | 20 | -57 |
| 78 | Motor_Thickness | -0.0035 | [-0.0115, +0.0035] | 5% | 12% | +0.0268 | 15 | -63 |
| 79 | Parietal_Thickness | -0.0036 | [-0.0104, +0.0019] | 2% | 6% | +0.0254 | 21 | -58 |
| 80 | Motor_L_Thickness | -0.0037 | [-0.0119, +0.0024] | 2% | 9% | +0.0267 | 16 | -64 |
| 81 | Cingulate_R_Thickness | -0.0038 | [-0.0113, +0.0026] | 4% | 10% | +0.0267 | 18 | -63 |

*Abbreviations: CI, confidence interval; MoCA, Montreal Cognitive Assessment; NP3*, Movement Disorder Society-Unified Parkinson's Disease Rating Scale Part III items; PPMI, Parkinson's Progression Markers Initiative; SBR, specific binding ratio (DAT-SPECT); SCOPA-AUT, Scales for Outcomes in Parkinson's Disease – Autonomic; UPDRS3, Movement Disorder Society-Unified Parkinson's Disease Rating Scale Part III total; UPSIT, University of Pennsylvania Smell Identification Test.*
