# Supplementary material for: Measurement fidelity, not model complexity, governs individual cognitive trajectory prediction in Parkinson's disease
Source: NPJ Digit Med. 2026 Jun 6;9:698. doi: 10.1038/s41746-026-02681-8 (PMC13572477; doi:10.1038/s41746-026-02681-8)
Supplement: Supplementary file 4 [file 41746_2026_2681_MOESM6_ESM.docx]

**Supplementary Figures S1–S2**

*Captions accompanying Supplementary Figure S1 (bootstrap permutation importance) and Supplementary Figure S2 (cohort propensity overlap). Image files supplied alongside this document as Supplementary_Figure_S1.png and Supplementary_Figure_S2.png.*

**Supplementary Figure S1.** Bootstrap permutation feature-importance stability for the cognitive trajectory target. Random Forest classifier with 200 bootstrap resamples on the PPMI discovery cohort (n = 1,018). Bars show the bootstrap-mean permutation importance for the top-ranked features under bootstrap aggregation; whiskers are 95% bootstrap confidence intervals. baseline_moca_total is the only feature with a top-10 inclusion frequency of 100%; its bootstrap mean importance (0.0253, 95% CI [0.0136, 0.0397]) is approximately three-fold larger than the next most stable imaging feature. Spearman rank correlation between the bootstrap-aggregated and the original single-fit importance profiles is rho = -0.034 (p = 0.76; n = 81 features), confirming that the overall importance ranking is not robust under perturbation. See Supplementary Table S3 for the full per-feature ranking.

**Supplementary Figure S2.** Cohort separability between PPMI and NACC. Histogram of estimated propensity scores from a logistic regression model trained to discriminate cohort membership (PPMI vs NACC Parkinson disease participants) using overlapping baseline variables (age, sex, education, baseline MoCA total). Combined sample n = 1,827. Cohort discrimination AUC = 0.715 ± 0.038 (5-fold cross-validation), indicating moderate but incomplete cohort separability; substantial overlap remains in regions where both cohorts share probability mass, supporting cross-cohort generalization analyses with the caveat of residual covariate shift in age and baseline cognition.
